# Relationships between lipid profiles and metabolic syndrome, insulin resistance and serum high molecular adiponectin in Japanese community-dwelling adults

**DOI:** 10.1186/1476-511X-10-79

**Published:** 2011-05-17

**Authors:** Ryuichi Kawamoto, Yasuharu Tabara, Katsuhiko Kohara, Tetsuro Miki, Tomo Kusunoki, Shuzo Takayama, Masanori Abe, Tateaki Katoh, Nobuyuki Ohtsuka

**Affiliations:** 1 Department of Community Medicine, Ehime University Graduate School of Medicine, Ehime 791-0295, Japan; 2 Department of Geriatric Medicine, Ehime University Graduate School of Medicine, Ehime 791-0295, Japan; 3 Department of Internal Medicine, Seiyo Municipal Nomura Hospital, Ehime 797-1212, Japan

## Abstract

**Background:**

There are few studies to demonstrate the associations between newly addressed lipid profiles and metabolic syndrome (MetS)-associated variables.

**Methods:**

Study participants without medications for hypertension, diabetes, or dyslipidemia {614 men aged 58 ± 14 (mean ± standard deviation; range, 20-89) years and 779 women aged 60 ± 12 (range, 21-88) years} were randomly recruited from a single community at the time of their annual health examination. The association between lipid profiles (total cholesterol (T-C), triglycerides (TG), low-density lipoprotein cholesterol (LDL-C), high-density lipoprotein cholesterol (HDL-C), non-HDL-C, T-C/HDL-C, TG/HDL-C, LDL-C/HDL-C ratio and MetS, Insulin resistance by homeostasis model assessment of insulin resistance (HOMA-IR), and serum HMW adiponectin were analyzed.

**Results:**

In multiple linear regression analysis, TG/HDL-C and T-C/HDL-C ratios as well as TG showed significantly strong associations with all three MetS-associated variables in both men and women. In men, the ROC curve analyses showed that the best marker for these variables was TG/HDL-C ratio, with the AUC for presence of MetS (AUC, 0.82; 95% CI, 0.77-0.87), HOMA-IR (AUC, 0.75; 95% CI, 0.70-0.80), and serum HMW adiponectin (AUC, 0.67; 95% CI, 0.63-0.71), respectively. The T-C/HDL-C ratio, TG, HDL-C, LDL-C/HDL-C ratio, and non-HDL-C also discriminated these markers; however all their AUC estimates were lower than TG/HDL-C ratio. These results were similar in women.

**Conclusion:**

In Japanese community-dwelling adults, lipid ratios of TG/HDL-C, T-C/HDL-C, LDL-C/HDL-C as well as TG and HDL-C were consistently associated with MetS, insulin resistance and serum HMW adiponectin. Lipid ratios may be used as reliable markers.

## Background

Metabolic syndrome (MetS) known as a clustering of cardiovascular risk factors associated with insulin resistance, hypertension, glucose intolerance, hypertriglyceridemia [1,2], and low levels of high-density lipoprotein cholesterol (HDL-C), is a major worldwide public health problem. In Japan also, MetS is quite common, affecting 13.3% to 25.3% of Japanese men [3,4] and may become even more common in the future with the continuous increase in the prevalence of obesity. Several epidemiological studies have demonstrated that MetS increases the risk of various cardiovascular diseases (CVD) [5] and diabetes [6].

Serum adiponectin, which is a 247-amino acid protein secreted specifically by adipose tissue, contains four differentiable domains that regulate lipid metabolism, glucose metabolism, and insulin sensitivity [7], and low circulating levels of serum adiponectin has been reported as a risk factor for the development of metabolic syndrome [8] and CVD [9]. The high molecular weight (HMW) form binds most avidly to its receptors and is one of the important molecules activating metabolic role of adiponectin [10].

Dyslipidemia is another well-known risk factor for CVD, as well as a component of MetS, and the role of HDL-C, triglycerides (TG) and low-density lipoprotein cholesterol (LDL-C) are already established as predictors of CVD [11]. On the other hand, a characteristic dyslipidemia is also associated with insulin resistance [12]. Several studies have reported the possibility that newly addressed lipid profiles might be more useful than the traditional ones used for CVD prediction, and measuring these variables might help identify insulin resistance and CVD [13]. Total cholesterol (T-C)/HDL-C, TG/HDL-C, and LDL-C/HDL-C ratio [13-15], as well as TG and HDL-C [16] are independently associated with insulin resistance and risk factors of CVD. However, in Japanese community-dwelling persons, there are few studies to demonstrate the associations between these lipid profiles, especially the lipid ratios, with MetS, insulin resistance and serum HMW adiponectin.

We took advantage of the large representative sample of Japanese adults who participated at the time of their annual health examination. We investigated how lipid profiles were associated with MetS, insulin resistance and serum HMW adiponectin in healthy Japanese adults. For this, we used cross-sectional data from community-dwelling participants without medication and clinical diabetes.

## Methods

### Subjects

Participants were recruited at the time of their annual health examination. The sample population compromised 3,164 middle-aged to elderly residents. Information on medical history, present conditions, and drugs were obtained by interview. Other characteristics, e.g., smoking and alcohol habits, and medication, were investigated by individual interviews using a structured questionnaire. Subjects taking medications for hypertension, diabetes, or dyslipidemia were excluded. The final study sample included 614 men and 779 women. This study was approved by the ethics committee of Ehime University School of Medicine, and written informed consent was obtained from each subject.

### Evaluation of confounding factors

Information on demographic characteristics and risk factors was collected using clinical files. Body mass index (BMI) was calculated by dividing weight (in kilograms) by the square of the height (in meters). We measured blood pressure in the right upper arm of participants in a sedentary position using an automatic oscillometric blood pressure recorder (BP-103i; Colin, Aichi, Japan) while the subjects were seated after having rested for at least 5 min. Smoking status was defined as the number of cigarette packs per day multiplied by the number of years smoked (pack · year), and the participants were classified into never smokers, past smokers, light smokers (<30 pack · year) and heavy smokers (≥30 pack · year). The daily alcohol consumption was measured using the Japanese liquor unit in which a unit corresponds to 22.9 g of ethanol, and the participants were classified into never drinkers, occasional drinkers (<1 unit/day), light drinkers (1-1.9 unit/day), and heavy drinkers (≥2 unit/day). T-C, TG, HDL-C, fasting blood glucose (FBG), creatinine (enzymatic method), immunoreactive insulin (IRI), and serum HMW adiponectin (FUJIREBIO, Tokyo, Japan) were measured during fasting. LDL-C levels were calculated using the Friedewald formula [17]. Participants with TG levels ≥400 mg/dl were excluded. Homeostasis model assessment of insulin resistance (HOMA-IR) was calculated from FBG and IRI levels using the following formula; {FBG (mg/dL) × IRI (mU/mL)}/405 [18].

### Metabolic Syndrome

We applied condition-specific cutoff points for MetS based on the modified guidelines for the diagnosis of MetS in Japan [19]. Metabolic syndrome was defined as obesity with at least two of the following three conditions: hypertension, dyslipidemia, and impaired fasting glucose (IFG). Obesity was defined as a BMI of ≥25.0 kg/m^2 ^[20]. Hypertension was defined as systolic blood pressure (SBP) ≥130 mmHg and diastolic blood pressure (DBP) ≥85 mmHg. Dyslipidemia was defined as TG concentrations ≥150 mg/dL and low HDL cholesterolemia (HDL-C <40 mg/dL). IFG was defined as a FBG level ≥110 mg/dL.

### Statistical analysis

Statistical analysis was performed using PASW Statistics 17.0 (Statistical Package for Social Science Japan, Inc., Tokyo, Japan). All values are expressed as mean ± standard deviation (SD), unless otherwise specified. Data for TG, FBG, HOMA-IR, and serum HMW adiponectin were skewed, and log-transformed for analysis. Differences between the two groups were determined by Student's t-test and χ^2 ^test. Subjects were divided into four groups based on the quartile of HOMA-IR and serum HMW adiponectin levels and the cutoff points for metabolic disorder were defined as the fourth quartile of HOMA-IR and first quartile of serum HMW adiponectin levels, respectively. Multiple linear regression analysis was used to evaluate the contribution of each lipid profile for the number of MetS components, HOMA-IR, and serum HMW adiponectin. In addition, areas under the receiver operating characteristic (ROC) curves were determined for each variable to identify the predictors of MetS-associated variables. The area under the curve (AUC) of ROC curves is a summary of the overall diagnostic accuracy of the test. The best markers have ROC curves that are shifted to the left with areas under the curve near unity. Nondiagnostic markers are represented by diagonals with the AUC of ROC curves close to 0.5. A value of *P *< 0.05 was considered significant.

## Results

### Background factors of subjects categorized by sex

Table 1 shows the value of each background factor categorized by sex. The subjects comprised 614 men aged 58 ± 14 (range, 20-89) years and 779 women aged 60 ± 12 (range, 21-88) years. The mean BMI was 23.3 ± 3.0 kg/m^2 ^and 22.9 ± 3.2 kg/m^2 ^in men and women, respectively. BMI, smoking status, alcohol consumption, systolic blood pressure (SBP), diastolic blood pressure (DBP), TG, and TG/HDL-C ratio were significantly higher but age, T-C, HDL-C, LDL-C, and Non-HDL-C were significantly lower in men than in women. There were no inter-group differences regarding T-C/HDL-C ratio, LDL-C/HDL-C ratio, eGFR, and prevalence of CVD.

**Table 1:** Characteristics of subjects categorized by sex

Characteristics	Men N = 614	Women N = 779	*P *-value*
Age (years)	58 ± 14	60 ± 12	0.001
Body mass index (kg/m^2^)	23.3 ± 3.0	22.9 ± 3.2	0.027
Smoking status {never/ex/light/heavy (%)}	37.8/20.8/17.9/23.5	97.2/0.8/1.9/0.1	<0.001
Alcohol consumption {never/light/moderate/heavy (%))	12.2/29.5/34.7/23.6	60.1/33.4/5.9/0.6	<0.001
Systolic blood pressure (mmHg)	135 ± 19	132 ± 22	0.005
Diastolic blood pressure (mmHg)	82 ± 11	78 ± 12	<0.001
Total cholesterol (mg/dL)	191 ± 34	209 ± 33	<0.001
Triglycerides (mg/dL)	97 (71-140)	85 (63-115)	<0.001
HDL cholesterol (mg/dL)	58 ± 14	65 ± 15	<0.001
LDL cholesterol (mg/dL)	109 ± 32	124 ± 30	<0.001
Non-HDL cholesterol (mg/dL)	132 ± 34	143 ± 33	<0.001
Total cholesterol/HDL cholesterol ratio	3.45 ± 1.01	3.35 ± 0.92	0.066
Triglycerides/HDL cholesterol ratio	1.67 (1.16-2.74)	1.29 (0.89-1.97)	0.001
LDL cholesterol/HDL cholesterol ratio	2.00 ± 0.81	2.02 ± 0.75	0.601
eGFR	83.9 ± 16.3	82.7 ± 16.5	0.183
Cardiovascular disease, %	5.7	3.9	0.124

HDL, high-density lipoprotein; LDL, low-density lipoprotein; eGFR, estimated glomerular filtration rate. Data presented are mean ± standard deviation. Data for triglycerides and triglycerides/HDL cholesterol ratio were skewed and were presented as median (interquartile range). Data for triglycerides and triglycerides/HDL cholesterol ratio were log-transformed for analysis. * Student's t-test or χ^2 ^test.

### Insulin resistance of subjects categorized by sex

Prevalence of MetS and  FBG were significantly higher in men than in women, but IRI, HOMA-IR and serum HMW adiponectin were significantly lower in men (Table 2).

**Table 2:** Insulin resistance and prevalence of metabolic syndrome of subjects categorized by sex

Characteristics	Men N = 614	Women N = 779	*P *-value*
Metabolic syndrome, %	14.2	8.3	0.001
Fasting blood glucose (mg/dL)	94 (89-101)	91 (86-97)	<0.001
Immuno-reactive insulin (mU/mL)	4.4 (2.8-7.2)	5.6 (3.8-8.0)	<0.001
HOMA-IR§	1.05 (0.64-1.81)	1.27 (0.84-1.84)	<0.001
Serum HMW adiponectin (μg/mL)	3.20 (1.97-4.92)	6.65 (4.42-9.64)	<0.001

HOMA-IR, homeostasis model assessment of insulin resistance; HMW, high molecular weight. Data for fasting blood glucose, fasting insulin, HOMA-IR, and serum HMW adiponectin were skewed and presented as median (interquartile range) values. §HOMA-IR was calculated using the following formula; {fasting blood glucose (FBG) (mg/dL) X fasting insulin (mU/mL)}/405. Data for fasting blood glucose, immuno-reactive insulin, HOMA-IR, and serum HMW adiponectin were log-transformed for analysis. * Student's t-test or χ^2 ^test.

### Synergistic effect of increased HOMA-IR and reduced serum HMW adiponectin

**Figure 1:**
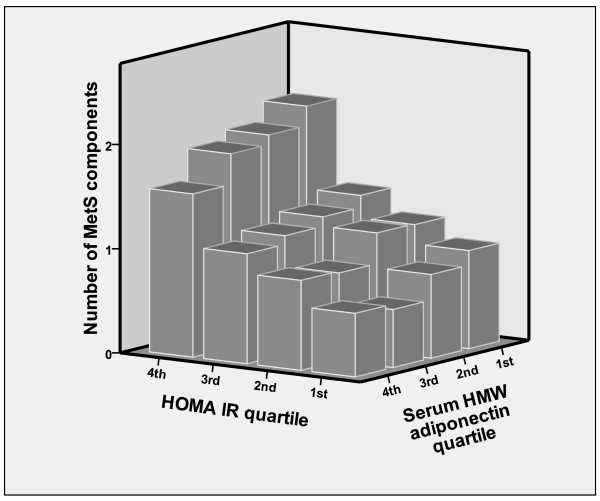
**Synergistic effect of increased HOMA-IR (homeostasis model assessment of insulin resistance) and reduced serum HMW (high molecular weight) adiponectin**. Mean accumulated number of the following MetS (metabolic syndrome) components: obesity, increased Blood pressure, impaired fasting glucose, dyslipidemia (hypertriglyceridemia or low high-density lipoprotein cholesterolemia). Study participants were divided into four groups (quartile) according to HOMA-IR and serum HMW adiponectin levels. Each quartile was calculated within sexes and then combined to eliminate any gender differences. Statistical significance was assessed by a general linear model for the following confounding factors: age, sex, BMI, smoking status, alcohol consumption, SBP, DBP, and eGFR.

Figure 1 showed synergistic effect of increased HOMA-IR and reduced serum HMW adiponectin. Mean accumulated number of MetS components was the highest in the highest HOMA-IR-lowest serum HMW adiponectin quartile group and lowest in the lowest HOMA-IR-highest serum HMW adiponectin quartile group, while HOMA-IR appears to be a more dominant determinant for MetS components than serum HMW adiponectin. HOMA-IR and serum HMW adiponectin were independently associated with the number of MetS components. We assessed the statistical significance of the relationship using a general linear model with the following confounding factors: age, sex, BMI, smoking status, alcohol consumption, SBP, DBP, and eGFR. The interaction between increased HOMA-IR and reduced serum HMW adiponectin was a significant and dependent determinant for the accumulated number of MetS components (F = 18.7, *P *< 0.001), in addition to their direct association (HOMA-IR, F = 77.0, *P *< 0.001; serum HMW adiponectin, F = 9.71, *P *= 0.002).

### Relationship between various risk factors including lipid profiles and MetS-associated variables categorized by sex

To further investigate whether lipid profiles can explain the MetS-associated variables independent of other known confounding factors, multiple linear regression analyses for the number of MetS components, HOMA-IR, and serum HMW adiponectin were performed (Table 3). In both men and women, TG/HDL-C and T-C/HDL-C ratios as well as TG showed significantly strong associations with all 3 of the MetS-associated variables. Non-HDL-C and LDL-C/HDL-C ratio also showed significant correlations; however almost their *β *estimates were lower than the TG/HDL-C ratio.

**Table 3:** Multiple linear regression analysis of the lipid measures with the number of MetS components, HOMA-IR and serum HMW adiponectin of subjects categorized by sex

Characteristics	*β*(*P*-value)					
	
	Number. of MetS components		HOMA-IR§		Serum HMW adiponectin	
	Men	Women	Men	Women	Men	Women
	N = 614	N = 779	N = 614	N = 779	N = 614	N = 779
Triglycerides (mg/dL)	0.337 (<0.001)	0.266 (<0.001)	0.237 (<0.001)	0.231 (<0.001)	-0.232 (<0.001)	-0.237 (<0.001)
HDL cholesterol (mg/dL)	-0.193 (<0.001)	-0.121 (<0.001)	-0.175 (<0.001)	-0.122 (<0.001)	0.218 (<0.001)	0.329 (<0.001)
LDL cholesterol (mg/dL)	-0.021 (0.503)	0.051 (0.048)	0.091 (0.009)	0.107 (0.001)	-0.126 (0.001)	-0.176 (0.001)
Non-HDL cholesterol (mg/dL)	0.115 (<0.001)	0.134 (<0.001)	0.164 (<0.001)	0.158 (<0.001)	-0.188 (<0.001)	-0.227 (<0.001)
Total cholesterol/HDL cholesterol ratio	0.256 (<0.001)	0.201 (<0.001)	0.252 (<0.001)	0.198 (<0.001)	-0.295 (<0.001)	-0.362 (<0.001)
Triglycerides/HDL cholesterol ratio	0.354 (<0.001)	0.257 (<0.001)	0.258 (<0.001)	0.228 (<0.001)	-0.270 (<0.001)	-0.315 (<0.001)
LDL cholesterol/HDL cholesterol ratio	0.143 (<0.001)	0.148 (<0.001)	0.209 (<0.001)	0.174 (<0.001)	-0.265 (<0.001)	-0.352 (<0.001)

MetS, Metabolic syndrome; HOMA-IR, homeostasis model assessment of insulin resistance; HMW, high molecular weight; HDL, high-density lipoprotein; LDL, low-density lipoprotein. §HOMA-IR was calculated using the following formula; {fasting blood glucose (mg/dL) X immuno-reactive insulin (mU/mL)}/405. Adjusted for age were BMI, smoking status, alcohol consumption, SBP, DBP, and eGFR. Data for triglycerides, triglycerides/HDL cholesterol ratio, and HOMA-IR were skewed, and log-transformed for analysis.

### Comparison of areas under ROC curves (95% CI) for potential markers of MetS-associated variables of subjects categorized by sex

In men, the ROC curve analyses showed that the best marker of the MetS-associated variables (Presence of MetS, HOMA-IR§ >1.98, and serum HMW adiponectin <2.97 μg/mL) was TG/HDL-C ratio, with the AUC for presence of MetS (AUC, 0.82; 95% CI, 0.77-0.87), HOMA-IR (AUC, 0.75; 95% CI, 0.70-0.80), and serum HMW adiponectin (AUC, 0.67; 95% CI, 0.63-0.71), respectively (Table 4). The T-C/HDL-C ratio, TG, HDL-C, non-HDL-C, and LDL-C/HDL-C ratio also discriminated these markers; however all their AUC estimates were lower than the TG/HDL-C ratio. These results were similar in women. On the other hand, each AUC of LDL-C for these three variables was near 0.50 in both women and men.

**Table 4:** Comparison of areas under the ROC curves (95% CI) for potential markers of insulin resistance of subjects categorized by sex

	Presence of MetS		HOMA-IR§ >1.98		Serum HMW adiponectin <2.97 μg/mL	
Characteristics	AUC (95% CI)	*P*-value	AUC (95% CI)	*P*-value	AUC (95% CI)	*P*-value
Men, N = 614						
Triglycerides (mg/dL)	0.81 (0.76-0.86)	<0.001	0.73 (0.68-0.78)	<0.001	0.67 (0.63-0.71)	<0.001
HDL cholesterol (mg/dL)	0.28 (0.22-0.34)	<0.001	0.31 (0.26-0.36)	<0.001	0.40 (0.35-0.44)	<0.001
LDL cholesterol (mg/dL)	0.56 (0.50-0.63)	<0.001	0.60 (0.54-0.65)	<0.001	0.51 (0.47-0.56)	0.577
Non-HDL cholesterol (mg/dL)	0.68 (0.62-0.74)	<0.001	0.66 (0.61-0.71)	<0.001	0.56 (0.51-0.61)	0.011
Total cholesterol/HDL cholesterol ratio	0.76 (0.70-0.81)	<0.001	0.73 (0.68-0.78)	<0.001	0.61 (0.56-0.65)	<0.001
Triglycerides/HDL cholesterol ratio	0.82 (0.77-0.87)	<0.001	0.75 (0.70-0.80)	<0.001	0.67 (0.63-0.71)	<0.001
LDL-cholesterol/HDL-cholesterol ratio	0.68 (0.62-0.74)	<0.001	0.69 (0.63-0.74)	<0.001	0.57 (0.53-0.62)	0.002
Women, N = 779						
Triglycerides (mg/dL)	0.83 (0.77-0.89)	<0.001	0.68 (0.64-0.73)	<0.001	0.63 (0.56-0.70)	<0.001
HDL cholesterol (mg/dL)	0.25 (0.19-0.32)	<0.001	0.38 (0.29-0.39)	<0.001	0.28 (0.22-0.34)	<0.001
LDL cholesterol (mg/dL)	0.62 (0.54-0.69)	0.021	0.60 (0.56-0.65)	<0.001	0.54 (0.47-0.62)	0.218
Non-HDL cholesterol (mg/dL)	0.70 (0.63-0.78)	0.457	0.64 (0.59-0.69)	<0.001	0.59 (0.52-0.66)	0.014
Total cholesterol/HDL cholesterol ratio	0.78 (0.72-0.84)	<0.001	0.69 (0.65-0.74)	<0.001	0.70 (0.63-0.76)	<0.001
Triglycerides/HDL cholesterol ratio	0.84 (0.78-0.89)	<0.001	0.70 (0.65-0.74)	<0.001	0.69 (0.62-0.75)	<0.001
LDL cholesterol/HDL cholesterol ratio	0.74 (0.68-0.81)	<0.001	0.68 (0.63-0.72)	<0.001	0.68 (0.62-0.75)	<0.001

ROC, receiver operating characteristics; MetS, metabolic syndrome; HOMA-IR, homeostasis model assessment of insulin resistance; HMW, high molecular weight; AUC, area under ROC curve; CI, confidence interval; HDL, high-density lipoprotein; LDL, low-density lipoprotein. §HOMA-IR was calculated using the following formula; {fasting blood glucose (mg/dL) X immuno-reactive insulin (mU/mL)}/405. Insulin resistance was defined as values exceeding the fourth quartile of HOMA-IR, and less than the first quartile of serum HMW adiponectin, respectively.

## Discussion

In the present study, we examined whether lipid profiles (i.e., TG, HDL-C, LDL-C, non-HDL-C, T-C/HDL-C ratio, TG/HDL-C ratio, and LDL-C/HDL-C ratio) were associated with MetS-associated variables (i.e., MetS, HOMA-IR, and serum HMW adiponectin) in Japanese community-dwelling adults. We demonstrated that HOMA-IR and serum HMW adiponectin, an active form of adiponectin, synergistically reflected the accumulated number of MetS components, and TG/HDL-C and T-C/HDL-C ratios, moreover, TG showed significantly and independently strong associations with these MetS-associated variables in both men and women. The best marker of three MetS-associated variables was TG/HDL-C ratio.

Resistance to insulin-mediated glucose disposal is distributed continuously through the general population [21], but we have no measurement which identify a participant with insulin resistance or insulin sensitive. Direct measurement of insulin resistance using the hyperinsulinemic-euglycemic clamp has practical limitations [22]. Some previous studies have shown that HOMA-IR based insulin resistance measurements have a strong correlation with glucose clamp-assessed insulin resistance [18,21]. Although these methods are less accurate than the hyperinsulinemic-euglycemic clamp, they serve as valuable surrogates for insulin resistance in normoglycemic individuals [23], but this limitation is mitigated when the number of subjects evaluated is large, as in our study [24].Then, we used fasting insulin and the HOMA-IR as markers of insulin resistance. In addition, serum adiponectin is also considered to be an important modulator of insulin sensitivity [25,26] and dyslipidemia [27]. Thus, we define the cut off value originally according to the level of the top quintile of HOMA-IR distribution and first quartile of serum HMW adiponectin in our subjects.

The presence of hypertriglyceridemia, low HDL-C concentrations, and high TG/HDL-C ratios almost never occurred as isolated disorders, and were nearly always associated with insulin resistance because insulin affects TG and HDL-C metabolism [28]. Previous studies have shown that several lipid ratios have been proposed as clinically simple and useful indicators of hyperinsulinemia or insulin resistance. The TG/HDL-C ratio have shown similar potential for insulin resistance, though the generalizability of this association has been not entirely consistent. In 50 white Americans, both TG and TG/HDL-C ratio were acceptable markers for insulin resistance, with area under the ROC curve of 0.763 and 0.770 [29], respectively, and in a study in an East African population, the TG/HDL-C ratio was found to be significantly associated with insulin resistance as measured by HOMA [30]. In contrast, recent studies have reported that theTG/HDL-C ratio was not significantly associated with insulin resistance in black adults [31] and adolescents [32]. The relationship between TG and TG/HDL-C with insulin resistance might be shown to differs by ethnicity. In our Japanese study, both TG/HDL-C and TC/HDL-C ratios as well as TG were useful makers of MetS-associated variables in both men and women, especially TG/HDL-C ratio in men. However, LDL-C was a weaker association with these variables. The present study provides additional information about lipid measures in both male and female Japanese.

Other lipid profiles have also shown their own relationship value for MetS. Kimm et al. [33] demonstrated that the lipid ratios of TC/HDL-C, LDL-C/HDL-C and TG/HDL-C, as well as TG and HDL-C, were each consistently associated with the number of metabolic syndrome components, insulin resistance quartiles (based on homeostatic model assessment), and log-transformed adiponectin quartiles. The lipids ratios that include information on at least two measures might have a more integrated explanation than single lipid measures such as TG or HDL-C [33]. In our study, the comparable results were seen.

Some limitations of this study must be considered. First, the response rate was as low as 35% that is usually the case in other conventional community studies in Japan. However, the relatively large sample size enabled the assessment of an extensive array of MetS-associated variables in relation to lipid profiles. Second, the cross-sectional study design is limited in its ability to eliminate causal relationships between lipid profiles and MetS-associated variables. Third, our definition of HOMA-IR is based on a single assessment of BS and IRI, which may introduce misclassification bias. Fourth, we used BMI ≥25 kg/m^2^ to classify individuals with visceral obesity because waist circumference measurements were not available, which might have caused an under estimation of the effect of visceral obesity on MetS [34]. Therefore the demographics and referral source may limit generalizability.

In conclusion, the present study demonstrated that special lipid profiles are associated with MetS-associated variables in a general population. The ability to identify who have MetS could help health care professionals in bringing about lifestyle interventions. In that context, use of LDL-C/HDL-C ratio or TG/HDL-C ratio described in this report is simple and useful. Further prospective population-based studies are needed to investigate the changes in lipid metabolism by lifestyle interventions.

## Competing interests

The authors declare that they have no competing interests.

## Authors' contributions

RK, YT, and KK participated in the design of the study, performed the statistical analysis and drafted the manuscript. NO, ToK, and TaK contributed to acquisition of data and its interpretation. ST and MA contributed to conception and design of the statistical analysis. TM conceived of the study, participated in its design, coordination and helped to draft the manuscript. All authors read and approved the manuscript.
